# *Rickettsia rickettsii* and *Rickettsia typhi *in inhabitants from a rural community of southeast Mexico

**DOI:** 10.17843/rpmesp.2022.391.10519

**Published:** 2022-03-18

**Authors:** Marco Torres-Castro, Enrique Reyes-Novelo, Henry Noh-Pech, Sokani Sánchez-Montes, Pablo Colunga-Salas, César Lugo-Caballero, Roger Iván Rodríguez-Vivas

**Affiliations:** 1 Universidad Autónoma de Yucatán, Mérida, México. Universidad Autónoma de Yucatán Universidad Autónoma de Yucatán Mérida Mexico; 2 Universidad Veracruzana, Veracruz, México. Universidad Veracruzana Universidad Veracruzana Veracruz Mexico

*To the editor*. The genus Rickettsia includes bacteria of the phylum Proteobacteria, of subclass a-1 of the order Rickettsiales. Traditionally, this genus is divided into two major clades: the typhoid group (TG) which includes *Rickettsia typhi* and *Rickettsia prowazekii,* transmitted by fleas and lice, and the spotted fever group (SFG) which includes more than 20 species, including *Rickettsia rickettsii*, mainly transmitted by “hard” ticks. These bacteria are characterized by causing emergent or re-emergent infection (depending on the infecting species) in people and animals around the world, known as rickettsial diseases or rickettsiosis ^1^. In southeastern Mexico, several *Rickettsia* species have been reported in humans, animals, and ectoparasites of numerous species ^2^, making it an endemic area and a “hot spot” for these bacteria. The aim of this study was to describe the frequency of *Rickettsia *spp. DNA in asymptomatic inhabitants of a rural community in southeastern Mexico and to identify the species involved in the infection through the use of bioinformatic analysis.

This research was approved by the Research Ethics Committee of the Centro de Investigaciones Regionales “Dr. Hideyo Noguchi”, Universidad Autónoma de Yucatán (act: CEI- 007-2018). Additionally, we considered the statutes of the World Medical Assembly of Helsinki and the International Code of Medical Ethics for the collection of biological samples and safeguarding of personal data.

From March to April 2018, we worked with 128 (asymptomatic) inhabitants of Maxcanú, Yucatán, Mexico. For each participant, we collected peripheral blood (5 mL in adults, 3 mL in children) in BD-Vacutainer® tubes with anticoagulant (EDTA-K2) that were kept in a cooler with refrigerants for transfer to the laboratory. The samples were centrifuged (15,000 revolutions per minute for 15 minutes at 24 °C) to collect the white blood cell package that was used for total DNA extraction using the QIAamp DNA Mini Kit® (QIAGEN®), “DNA Purification from liquids and fluids” protocol. The extracted DNA samples were evaluated in a NanoDrop-2000® spectrophotometer (Thermo Scientific®) to determine their concentration and purity.

Two PCRs were performed to identify *Rickettsia* DNA in blood samples, one nested and one multiplex semi-nested, for detection of the *17kDa* and *sca5* genes, respectively, using the previously described methodologies ^3^. Positive (*Rickettsia conorii* DNA) and negative (sterile water) controls were considered in all reactions. Electrophoresis of the products of both PCRs was carried out on agarose gels (1%) stained with ethidium bromide (8%).

The *sca5* amplicons (*17kDa* amplicons were not considered) with the expected sizes (420 and 230 base pairs for *R. rickettsii* and *R. typhi*, respectively) were purified with the Gel DNA Recovery kit (ZymocleanTM®) according to standardized specifications, and sent to the DIMYGEN® laboratory (Merida, Mexico) for sequencing (sense and antisense) by the Sanger method. The obtained sequences were edited and aligned with MEGA version 7.0® software (https://www.megasoftware.net/), and subsequently compared, using the Megablast algorithm, with partial *sca5* sequences from *Rickettsia* previously deposited in GenBank (https://blast.ncbi.nlm.nih.gov/Blast.cgi), in order to determine their identity and coverage percentages.

*Rickettsia* DNA was identified in 34.4% (44/128) of the blood samples. The results of the GenBank analysis are shown in Table 1. As can be confirmed, seven sequences showed identity and coverage for *R. rickettsii* and two for *R. typhi*.

**Table 1:** Coverage and identity percentages shown by bioinformatic analysis with Megablast, homologous *Rickettsia* species and GenBank access number for homologous sequences with sequences obtained from *sca5* amplicons in an asymptomatic population from southeastern Mexico.

**Sequence identification code of the *sca5* amplicon sequences**	Identity and coverage % shown by Megablast	**Homologous species of *Rickettsia***	**GenBank access number for homologous sequences with sequences obtained from *sca5* amplicons.**
MaxSH020	99.75-100	*Rickettsia rickettsii*	CP018914.1, CP018913.1, CP006010.1, CP006009.1, CP000766.3
MaxSH024	99.13-100	*Rickettsia rickettsii*	CP018914.1, CP018913.1, CP006010.1, CP006009.1, CP000766.3
MaxSH028	99.7-100	*Rickettsia rickettsii*	CP018914.1, CP018913.1, CP006010.1, CP006009.1, CP000766.3
MaxSH030	99.12-100	*Rickettsia rickettsii*	CP018914.1, CP018913.1, CP006010.1, CP006009.1, CP000766.3
MaxSH032	92.49-100	*Rickettsia rickettsii*	CP018914.1, CP018913.1, CP006010.1, CP006009.1, CP000766.3
MaxSH036	96.66-100	*Rickettsia rickettsii*	CP018914.1, CP018913.1, CP006010.1, CP006009.1, CP000766.3
MaxSH037	99.75-100	*Rickettsia rickettsii*	CP018914.1, CP018913.1, CP006010.1, CP006009.1, CP000766.3
MaxSH085	91.26-97	*Rickettsia typhi*	LS992663.1, CP003398.1, CP003397.1, HQ236390.1, AE017197.1
MaxSH087	96.23-99	*Rickettsia typhi*	LS992663.1, CP003398.1, CP003397.1, HQ236390.1, AE017197.1

Isolated cases of infection with *R. typhi* ^4^ ^,^ ^5^ and *R. rickettsii* ^6^ have been reported in inhabitants and animals of Yucatan; nevertheless, our findings are important because of the evidence of infection (bacteremia) with two species of *Rickettsia* in the same asymptomatic studied population, which represents advances in the epidemiological study and clinical approach to cases of rickettsiosis in southeastern Mexico. Changes in the ecological niche, the continuous circulation of vectors (ticks and fleas), the presence of reservoirs (animals) and accidental hosts (humans) that maintain the transmission cycles of the different Rickettsia species ^2^ ^,^ ^3^ in Yucatan, could be associated with our report.

One of the limitations of our study is that only nine positive products were sent for sequencing, which could limit the identification of more *Rickettsia* species involved in the infection. Another limitation is the lack of clinical follow-up and subsequent generation of data from Rickettsia DNA-positive individuals. These data are relevant to improve the approach and diagnosis of rickettsial diseases in Yucatan.

Two *Rickettsia* species were found in asymptomatic individuals in southeastern Mexico. Active surveillance and vector control (ticks and fleas) are recommended to reduce the risk of *Rickettsia* transmission in the inhabitants and domestic animals of Yucatan communities ^3^, as well as to conduct studies to better understand the protagonists in the rickettsial cycle and reduce the incidence of these diseases in southeastern Mexico.
