# Supplementary material for: Rickettsia rickettsii and Rickettsia typhi in inhabitants from a rural community of southeast Mexico
Source: Rev Peru Med Exp Salud Publica. 2022 Mar 18;39(1):124–5. doi: 10.17843/rpmesp.2022.391.10519 (PMC11397770; doi:10.17843/rpmesp.2022.391.10519)
Supplement: Translation

# Translation: *Rickettsia rickettsii* y *Rickettsia typhi* en habitantes de una comunidad rural del sureste de México

**Authors:** Marco Torres-Castro, Enrique Reyes-Novelo, Henry Noh-Pech, Sokani Sánchez-Montes, Pablo Colunga-Salas, César Lugo-Caballero, Roger Iván Rodríguez-Vivas

**Affiliations:** 1 Universidad Autónoma de Yucatán, Mérida, México.; 2 Universidad Veracruzana, Veracruz, México.

Sr. Editor. El género *Rickettsia* incluye bacterias del phylum Proteobacteria, de la subclase a-1 del orden Rickettsiales. Tradicionalmente, este género se divide en dos grandes clados: el grupo del tifo (GT) que incluye a *Rickettsia typhi *y *Rickettsia prowazekii* transmitidas por pulgas y piojos, y el grupo de las fiebres manchadas (GFM) que incluye más de 20 especies, entre ellas *Rickettsia rickettsii*, principalmente transmitidas por garrapatas «duras». Estas bacterias se caracterizan por ocasionar infección emergente o reemergente (según la especie que infecta) en personas y animales alrededor del mundo, conocidas como enfermedades rickettsiales o rickettsiosis ^1^. En el sureste de México han sido reportadas varias especies de *Rickettsia* en personas, animales y ectoparásitos de numerosas especies ^2^, por lo que se considera una zona endémica y un «punto caliente» para estas bacterias. El objetivo del presente estudio fue describir la frecuencia de ADN de *Rickettsia* spp. en habitantes asintomáticos de una comunidad rural del sureste de México e identificar las especies involucradas en la infección a través del uso de análisis bioinformáticos.

Esta investigación fue aprobada por el Comité de Ética en Investigación del Centro de Investigaciones Regionales «Dr. Hideyo Noguchi», Universidad Autónoma de Yucatán (acta: CEI-007-2018). Adicionalmente, para la toma de muestras biológicas y resguardo de datos personales, se consideraron los estatutos de la Asamblea Médica Mundial de Helsinki y del Código Internacional de Ética Médica.

De marzo a abril de 2018 se trabajó con 128 habitantes (asintomáticos) de Maxcanú, Yucatán, México. A cada uno se les recolectó sangre periférica (5 mL en adultos, 3 mL en niños) en tubos BD-Vacutainer® con anticoagulante (EDTA-K2) y se conservó en una nevera con refrigerantes para su traslado al laboratorio. Las muestras se centrifugaron (15 000 revoluciones por minuto durante 15 minutos a 24 °C) para recolectar el paquete de glóbulos blancos que sirvió en la extracción de ADN total realizada con el kit QIAamp DNA Mini Kit® (QIAGEN®), protocolo «DNA Purification from liquits and fluids». Las muestras de ADN extraídos fueron evaluados en un espectrofotómetro NanoDrop-2000® (Thermo Scientific®) para determinar su concentración y pureza. 

Para identificar ADN de *Rickettsia *en las muestras sanguíneas se realizaron dos PCR, una anidada y otra semianidada múltiple, dirigidas a la detección de los genes *17kDa *y *sca5*, respectivamente, utilizando las metodologías descritas previamente ^3^. En todas las reacciones se consideraron controles positivos (ADN de *Rickettsia conorii*) y negativos (agua estéril). La electroforesis de los productos de ambas PCR se realizó en geles de agarosa (1%) teñidos con bromuro de etidio (8%).

Los amplificados de *sca5* (no se consideraron amplificados de *17kDa*) con los tamaños esperados (420 y 230 pares de bases para *R. rickettsii* y* R. typhi,* respectivamente) se purificaron con el kit Gel DNA Recovery (ZymocleanTM®) según las especificaciones estandarizadas, y se enviaron al laboratorio DIMYGEN® (Mérida, México) para su secuenciación (sentido y antisentido) por el método Sanger. Las secuencias obtenidas fueron editadas y alineadas con el *software* MEGA versión 7.0® (https://www.megasoftware.net/), y posteriormente comparadas, utilizando el algoritmo Megablast, con secuencias parciales de *sca5* pertenecientes a *Rickettsia*, previamente depositadas en GenBank (https://blast.ncbi.nlm.nih.gov/Blast.cgi), para conocer sus porcentajes de identidad y cobertura.

Se identificó ADN de *Rickettsia* en 34,4% (44/128) de las muestras sanguíneas. Los resultados del análisis en GenBank arrojaron lo indicado en la Tabla 1. Como puede confirmarse, siete secuencias presentaron identidad y cobertura para *R*. *rickettsii *y dos para *R*. *typhi*.

**Tabla 1:** Porcentajes de cobertura e identidad mostrados por el análisis bioinformático con Megablast, especies homólogas de *Rickettsia* y número de acceso de GenBank para las secuencias homólogas con las secuencias obtenidas de los amplificados de *sca* ^5^ en una población asintomática del sureste de México.

Código de identificación de las secuencias de los amplificados de *sca5*	% de identidad y cobertura mostrados por Megablast	**Especies homólogas de *Rickettsia***	**Número de acceso GenBank para las secuencias homólogas con las secuencias obtenidas de los amplificados de *sca5***
MaxSH020	99,75-100	*Rickettsia rickettsii*	CP018914.1, CP018913.1, CP006010.1, CP006009.1, CP000766.3
MaxSH024	99,13-100	*Rickettsia rickettsii*	CP018914.1, CP018913.1, CP006010.1, CP006009.1, CP000766.3
MaxSH028	99,7-100	*Rickettsia rickettsii*	CP018914.1, CP018913.1, CP006010.1, CP006009.1, CP000766.3
MaxSH030	99,12-100	*Rickettsia rickettsii*	CP018914.1, CP018913.1, CP006010.1, CP006009.1, CP000766.3
MaxSH032	92,49-100	*Rickettsia rickettsii*	CP018914.1, CP018913.1, CP006010.1, CP006009.1, CP000766.3
MaxSH036	96,66-100	*Rickettsia rickettsii*	CP018914.1, CP018913.1, CP006010.1, CP006009.1, CP000766.3
MaxSH037	99,75-100	*Rickettsia rickettsii*	CP018914.1, CP018913.1, CP006010.1, CP006009.1, CP000766.3
MaxSH085	91,26-97	*Rickettsia typhi*	LS992663.1, CP003398.1, CP003397.1, HQ236390.1, AE017197.1
MaxSH087	96,23-99	*Rickettsia typhi*	LS992663.1, CP003398.1, CP003397.1, HQ236390.1, AE017197.1

Se han reportado casos aislados de infección con *R*. *typhi* ^4^ ^,^ ^5^ y *R*. *rickettsii* ^6^ en habitantes y animales de Yucatán; no obstante, los hallazgos presentados son importantes debido a que se evidencia la infección (bacteriemia) con dos especies de *Rickettsia* en una misma población estudiada de asintomáticos, lo que representa avances en el estudio epidemiológico y el abordaje clínico de casos de rickettsiosis en el sureste de México. Los cambios en el nicho ecológico, la circulación continua de vectores (garrapatas y pulgas), la presencia de reservorios (animales) y hospederos accidentales (humanos) que mantienen los ciclos de transmisión de las distintas especies de *Rickettsia* ^2^ ^,^ ^3^ en Yucatán, podrían estar asociados al presente reporte. 

Dentro de las limitaciones del estudio puede mencionarse que se enviaron a secuenciación únicamente nueve productos positivos, lo cual pudo limitar la identificación de más especies de *Rickettsia* involucradas en la infección. Otra limitante es la falta de seguimiento clínico y la subsecuente generación de datos en las personas positivas a la presencia de ADN de *Rickettsia*. Estos datos son relevantes para mejorar el abordaje y diagnóstico de las rickettsiosis en Yucatán.

Se encontraron dos especies de *Rickettsia* en personas asintomáticas del sureste de México. Se recomienda realizar vigilancia activa y control de vectores (garrapatas y pulgas) para reducir el riesgo de transmisión de *Rickettsia *en los habitantes y animales domésticos de las comunidades de Yucatán ^3^, así como realizar estudios para un mejor conocimiento de los protagonistas del ciclo de las rickettsiosis y disminuir la incidencia de estas enfermedades en el sureste de México.
